# Pharmacodynamics of nine generic products of amikacin compared with the innovator in the neutropenic mouse thigh infection model

**DOI:** 10.1186/s13104-015-1507-z

**Published:** 2015-10-07

**Authors:** Andres F. Zuluaga, Carlos A. Rodriguez, Maria Agudelo, Omar Vesga

**Affiliations:** GRIPE [Grupo Investigador de Problemas en Enfermedades infecciosas], Universidad de Antioquia, Calle 70 No. 52-21, Medellín, Colombia; Department of Pharmacology and Toxicology at Medical School, Universidad de Antioquia, Calle 70 No. 52-21, Medellín, Colombia; Department of Internal Medicine, Universidad de Antioquia, Calle 70 No. 52-21, Medellín, Colombia; Infectious Diseases Unit, Hospital Universitario San Vicente Fundación, Medellín, Colombia

**Keywords:** Amikacin, Animal models, Generics, Therapeutic equivalence, Antimicrobial resistance

## Abstract

**Background:**

Previously, we validated the mouse thigh infection model to test the therapeutic equivalence of generic antibiotic products. Here, our aim was to compare the in vivo efficacy of amikacin products in clinical use in Colombia using this animal model.

**Results:**

All except one generic product had the same in vitro potency, judging by the lack of differences on MIC and MBC compared with the innovator. However, eight of nine generic products failed in the neutropenic mouse thigh infection model to achieve the innovator’s maximum effect (*E*_*max*_ ≤ 5.65 for the generics vs. 6.58 log_10_ CFU/g for the innovator) against *Escherichia coli* SIG-1, after subcutaneous treatment every 6 h with doses ranging from 1.5 to 3072 mg/kg per day.

**Conclusion:**

As we demonstrated previously with other antibiotics such as vancomycin, gentamicin and oxacillin, the generic products of amikacin failed the in vivo efficacy testing. The therapeutic equivalence should be assessed in vivo before clinical approval of generic products.

## Background

Generic substitution of medications is a common practice [1–3]. Worldwide, there is an abbreviated approval pathway for generic drugs of small molecules in which the comparative clinical trials are waived [4, 5], after demonstrating good manufacturing practices and bioequivalence in healthy volunteers [6, 7]. Furthermore, bioequivalence is waived for intravenous generics under the assumption that pharmaceutical equivalence predicts therapeutic equivalence accurately [8]. This approach has certainly rendered the desired economic results [9], but at the price of neglecting solid evidence documenting the clinical failure of intravenous generics of vancomycin and cefuroxime [10]. Besides, an animal infection model was validated by our group to determine the therapeutic equivalence of antimicrobials [11, 12], in which many generic products of vancomycin [13], oxacillin [14, 15], gentamicin [16], meropenem [17], lincomycin [18], ampicillin [19], and penicillin G [20] failed to kill the same number of microorganisms as the innovators. Of great concern, those generics of vancomycin that failed therapeutic equivalence selected the resistant subpopulation of *Staphylococcus aureus* [21], whilst therapeutically equivalent generics of ciprofloxacin were indistinguishable from the innovator in terms of selection of resistant *Pseudomonas aeruginosa* [22].

Amikacin is derived from kanamycin and its structure confers stability towards many enzymes, mainly from Gram negative bacteria, that hydrolyze other aminoglycosides [23]. This quality makes it the preferred aminoglycoside to prescribe along with a β-lactam to treat diverse nosocomial infections. During the execution of this study, the sudden discontinuation of the innovator product (Amikin^®^, Bristol Myers-Squibb) forced us to stop the in vivo comparative experiments. In view of the impossibility of obtaining additional data, we decided to publish the available evidence.

## Results

### Antibiotics

Table 1 lists the products tested with their pharmaceutical form, lot numbers, manufacturers and distributors. The demonstration of pharmaceutical equivalence for Carlon, Gencol, Pisa, Scalpi and Sigma generic products was published previously by our group [8]. Seven of nine generic products (78 %) were produced in Colombia while the other two (Genven and Pisa) were made in Venezuela and Mexico, respectively. The Farmionni-Lubelca consortium manufactured three (Scalpi, Serpharma, Zokumey) of the nine generics tested (33 %), but they were analyzed as independent products.

**Table 1:** General description of the amikacin products studied

Amikacin product	Form	Demonstrated pharmaceutical equivalence^a^	Batch	Manufacturer	Distributor
BMS (innovator)	1 g in 4 ml	Not applicable	99A106	Grove, Ecuador	The manufacturer
	0.5 g in 2 ml		00G030 05H091A	Bristol-Myers Squibb, Ecuador	
Carlon	0.5 g in 2 ml	Yes	111V0203	Carlon, Colombia	The manufacturer
FormasG	0.5 g in 2 ml	No	02525	Vitropharma, Colombia	Formas genericas farmaceuticas, Colombia
Gencol	0.25 g in 2 ml	Yes	0100 0200	Chalver, Colombia	The manufacturer
Genven	0.5 g in 2 ml	No	904037	Leti for Genven, Venezuela	The manufacturer
Pisa	0.5 g in 2 ml	Yes	060865 011306	PiSa, Mexico	ECAR, Colombia
Quimicol	0.5 g in 2 ml	No	3780199	Quimicol, Colombia	The manufacturer
Scalpi	0.5 g in 2 ml	Yes	AK030072	Farmionni-Lubelca, Colombia	Farmionni scalpi, Colombia
Serpharma	0.25 g in 2 ml	No	AK020086	Farmionni-Lubelca, Colombia	Serpharma, Colombia
	0.1 g in 2 ml		AK010172		
Sigma (reference)	1 g powder	Yes	120K1643	Sigma Chemical Co, USA	The manufacturer
Zokumey	0.25 g in 2 ml	No	AK020035	Farmionni-Lubelca, Colombia	Zokumey pharma, Colombia

^a^The pharmaceutical equivalence (same potency and concentration of the active ingredient) tested by microbiological assay was published elsewhere [8]

### Susceptibility testing

Table 2 shows the MIC and MBC of all products against *E. coli* SIG-1 or *P. aeruginosa* ATCC 27853. All but one generic amikacin product exhibited the same in vitro efficacy of the innovator; the exception was Serpharma, which MIC and MBC were 10- and 16-fold higher against both strains (P < 0.05 by Dunn’s multiple comparison test). These results were reproducible in assays performed in different days.

**Table 2:** Comparison of the in vitro biological potency of the amikacin products studied

Product	*Escherichia coli* SIG-1						*Pseudomonas aeruginosa* ATCC 27853					
	MIC	Min	Max	MBC	Min	Max	MIC	Min	Max	MBC	Min	Max
BMS	1.59	1.00	2.00	1.78	1.00	2.00	4.00	4.00	4.00	10.08	8.00	16.00
Carlon	4.00	4.00	4.00	8.00	8.00	8.00	5.66	4.00	8.00	5.66	4.00	8.00
FormasG	4.00	4.00	4.00	4.00	4.00	4.00	5.66	4.00	8.00	8.00	8.00	8.00
Gencol	2.00	2.00	2.00	2.00	2.00	2.00	4.00	4.00	4.00	8.00	4.00	16.00
Genven	2.00	2.00	2.00	2.00	2.00	2.00	2.83	2.00	4.00	5.66	4.00	8.00
Pisa	2.00	2.00	2.00	2.83	2.00	4.00	4.00	4.00	4.00	11.31	8.00	16.00
Quimicol	2.83	1.00	8.00	4.00	2.00	8.00	3.36	2.00	4.00	6.73	4.00	16.00
Scalpi	2.83	2.00	4.00	2.83	2.00	4.00	4.00	4.00	4.00	4.00	4.00	4.00
Serpharma**	19.03	16.00	32.00	22.63	16.00	32.00	38.05	32.00	64.00	64.00	64.00	64.00
Sigma	1.41	1.00	2.00	2.83	2.00	4.00	4.00	4.00	4.00	8.00	8.00	8.00
Zokumey	2.00	2.00	2.00	4.00	2.00	8.00	4.00	4.00	4.00	11.31	8.00	16.00

Concentrations are expressed as geometric mean and range (Min. and Max) in mg/L

*MIC* minimal inhibitory concentration, *MBC* minimal bactericidal concentration

** *p* value <0.05 by Dunn’s multiple comparison test

### Reliability of the animal model to test therapeutic equivalence

**Fig. 1:**
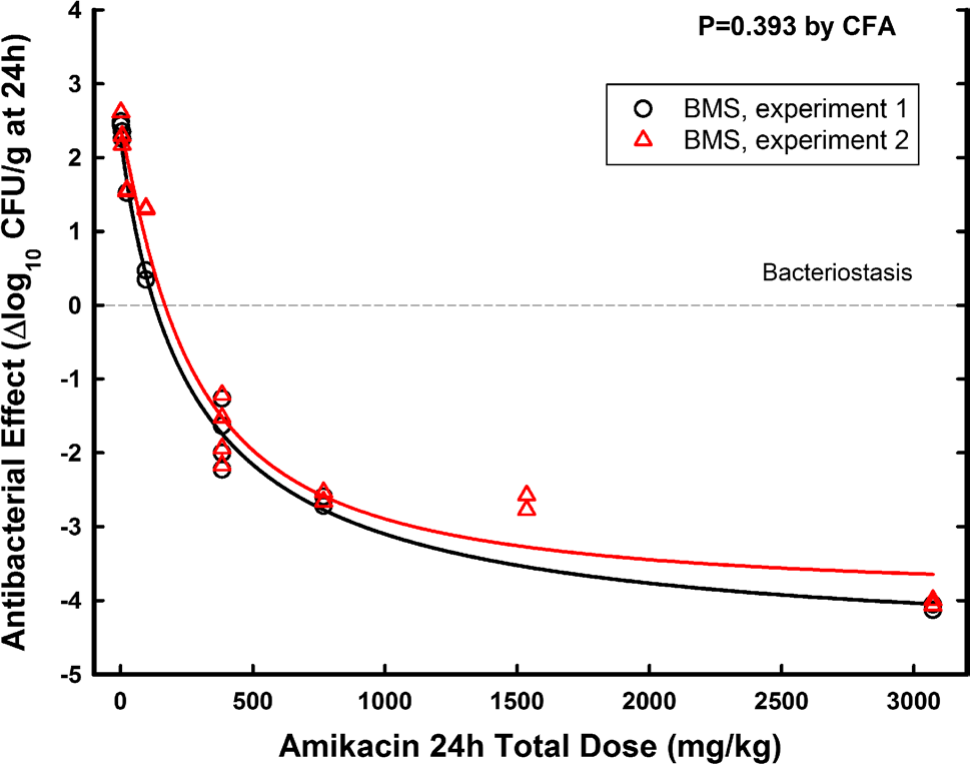
Reliability of the neutropenic murine thigh infection model with the innovator of amikacin (BMS) in two independent experiments. The non-significant P value (0.393) from global curve fitting analysis (CFA) indicates that the underlying populations are better described by a *single curve*, confirming the model’s reliability for testing therapeutic equivalence

The repeatability of the PD parameters was assessed in two different days with the same batch of the innovator (batch 99A106). Figure 1 shows that there was no difference in the non-linear regression (NLR) from two independent experiments (P = 0.39 by CFA) with innovator amikacin.

### Therapeutic equivalence testing

**Fig. 2:**
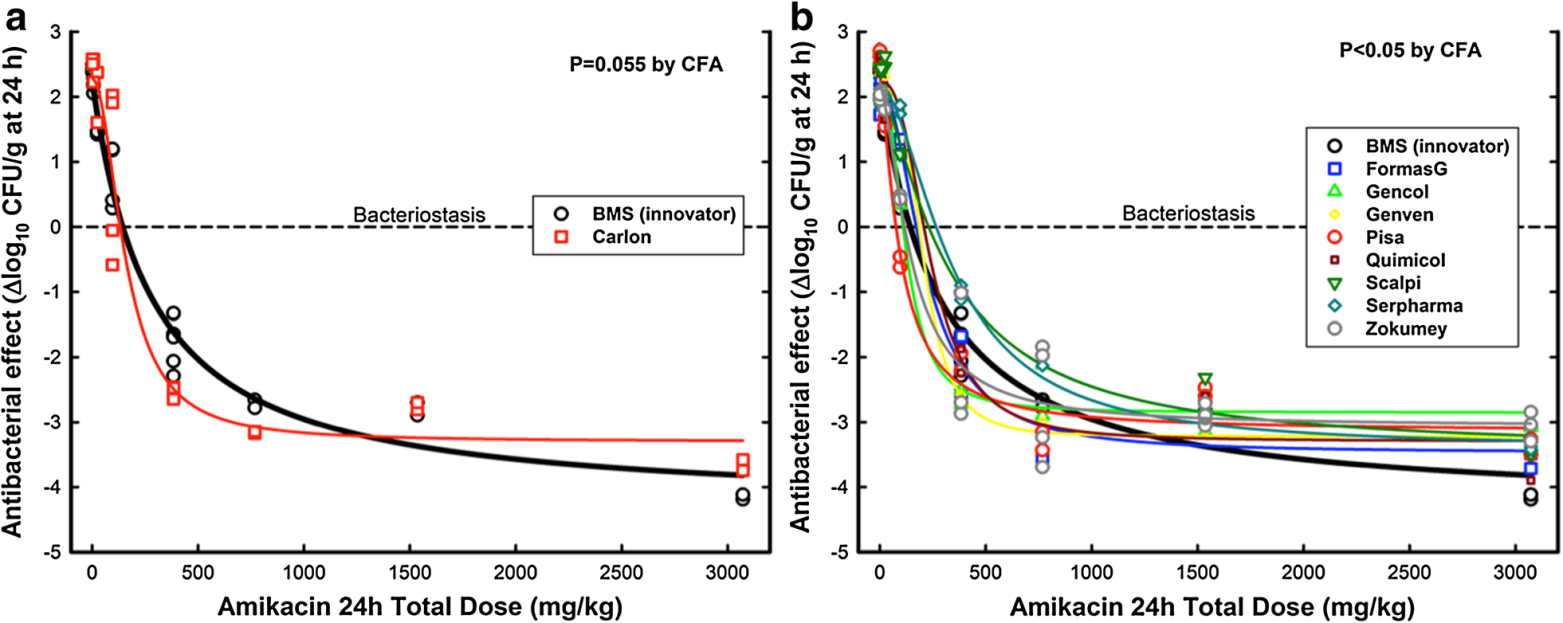
In *panel* **a**, the in vivo activity of the Carlon generic product of amikacin compared with the innovator (BMS). The non-significant P value of the curve-fitting analysis (0.055) indicates that the generic is therapeutically equivalent to the innovator, however the higher data dispersion reduced the power of the test to detect significant differences from 87 % to 63 %. In *panel* **b**, the in vivo activity of eight generic products of amikacin compared with the innovator (BMS). The global curve-fitting analysis (P < 0.05) demonstrates that the generics are described by independent *curves*, characterized by reduced *E*_*max*_ compared with the innovator (see Table 3), indicating that they lack therapeutic equivalence, despite similar in vitro activity

Untreated animals had 7.04–7.34 log_10_ CFU/g when treatment started (0 h) and 9.07–9.78 log_10_ CFU/g 24 h later when therapy was finished (net growth = 2.24 ± 0.29 log_10_ CFU/g). All products tested yielded valid non-linear regressions describing the dose–response relationships obtained by Hill’s Equation (Fig. 2). The PD parameters for the innovator were *E*_*max*_ = 6.58 ± 0.40 log_10_ CFU/g, *ED*_50_ = 272 ± 44.6 mg/kg per day, and *N* = 1.02 ± 0.12, while the magnitudes of primary (*E*_*max*_, *ED*_50_, *N*) and secondary (*BD*, *1LKD*, and *2LKD*) parameters of the other nine products are summarized in Table 3.

**Table 3:** In vivo pharmacodynamic parameters of nine generics and the innovator product of amikacin

Amikacin	AdjR^2^	S_y|x_	*E*_*max*_	SE	*ED*_50_	SE	*N*	SE	*BD*	SE	*1LKD*	SE	*2LKD*	SE	P value
															(CFA)
BMS (innovator)	0.97	0.37	6.58	0.40	272	44.6	1.02	0.12	144	12.7	266	19.9	490	40.8	NA
Carlon	0.93	0.65	5.58	0.33	160	27.0	2.01	0.49	132	17.3	190	28.6	288	55.8	0.055
FormasG	0.98	0.36	5.65	0.19	216	19.6	2.01	0.27	169	16.7	242	17.7	357	23.9	0.013
Gencol	0.99	0.24	5.10	0.14	122	11.0	2.26	0.43	110	8.30	157	15.5	248	37.1	0.001
Genven	1.00	0.17	5.36	0.07	209	13.2	3.35	0.31	185	11.9	232	13.6	301	17.8	<0.0001
Pisa	0.98	0.38	5.45	0.22	96	14.1	1.36	0.22	77	9.70	133	16.5	257	40.2	0.001
Quimicol	0.98	0.41	5.53	0.22	241	30.4	2.61	0.56	208	27.9	275	30.1	380	43.2	0.004
Scalpi	0.98	0.38	5.65	0.36	324	48.8	1.35	0.24	228	26.4	384	36.6	684	78.3	0.004
Serpharma	0.99	0.22	5.58	0.17	343	23.6	1.76	0.20	261	17.4	395	21.0	624	39.0	<0.0001
Zokumey	0.95	0.51	5.25	0.24	145	25.7	1.57	0.35	117	18.7	191	30.1	347	68.5	0.044

*AdjR* ^*2*^ adjusted coefficient of determination, *S*_*y|x*_ standard error of the estimate, *CFA* curve fitting analysis, *E*_*max*_ maximum effect, *SE* standard error, *ED*_*50*_ effective dose to kill 50 % of *E*_*max*_, *N* slope, *BD* bacteriostatic dose, *1LKD* and *2LKD* 1- and 2-log kill dose, respectively

Except for Carlon product (Fig. 2, panel a), the remaining eight generics failed to reach the innovator’s *E*_*max*_, which ranged from 5.10 to 5.65 log_10_ CFU/g; in the best case, it was one order of magnitude lower than the innovator. It means that the innovator killed ~3.80 million microorganisms per gram of tissue at the maximal total dose used, whilst the most effective generic killed only 0.45 million. Although two generics (Gencol and Pisa) had greater potency than the innovator comparing their bacteriostatic dose (≤110 ± 8.30 vs. 144 ± 12.7 mg/kg per day), both also had significantly lower *E*_*max*_ (P = 0.0003).

## Discussion

Here, our results with amikacin indicate that almost all generics (eight of nine products) failed therapeutic equivalence in a head-to-head in vivo comparison with the innovator, independently of their pharmaceutical equivalence. These data are similar to previous results with other antibiotics [13, 15, 17], reinforcing the idea that therapeutic equivalence of generic antimicrobials cannot be predicted from pharmaceutical equivalence or in vitro testing and therefore requires in vivo studies [11, 12].

The reliability of the thigh infection model to test the efficacy of antibiotics was assessed in two independent experiments with the innovator, exhibiting the same PD profile (Fig. 1). Besides, the similar in vivo lower efficacy of three generic products from the same manufacturer (all produced by Farmionni-Lubelca) confirmed the consistency of the model’s findings.

Generic drugs are necessary to regulate drug price. But the scant information provided in the abbreviated way used by generic manufacturers have arisen some theoretical concerns [10, 24, 25] that are experimentally supported by our results. In this context, the “contamination” of bioequivalent generic heparin with oversulfated chondroitin sulfate killed approximately 1000 patients around the world [26], but generic antibiotics may entail an even worse problem: antimicrobial resistance [27, 28]. We already demonstrated that so called “bioequivalent” generics of vancomycin devoid of therapeutic equivalence do enrich resistant subpopulations of *S. aureus* after exposure for only 12 days in the neutropenic murine thigh infection model [21]. In contrast, fully equivalent generic products of ciprofloxacin do not exhibit difference on resistance profile [22]. Here, similar to the vancomycin case, the therapeutically nonequivalent generics of amikacin do not sterilize the thighs even at the highest dose (3072 mg/kg per day), leaving at least 3 million bacterial cells per gram of tissue exposed to the antibiotic, but alive. Then, the risk of resistance is not a minor point [29].

The study by Miller et al. supports our hypothesis about the impact on resistance of the massive use of generic products of amikacin failing therapeutic equivalence [30]. They demonstrated that the aminoglycoside resistance mechanisms changed with the time (comparing studies before and after 1990's decade) and geographical region, according to the increased usage of these drugs. According to Miller et al., the baseline resistance level of *Citrobacter* spp., *Enterobacter* spp. or *Klebsiella* spp. to amikacin was lower than 20 % before 1987 (when generic consumption was limited), moment at which a continuous rise was observed reaching 60 % in countries like Chile, Uruguay, Mexico and Venezuela. They also found that it was precisely during this period of massive use of generic antimicrobials (after 1987), that new enzymes capable to degrade amikacin appeared, such as AAC(6′)-I alone or combined with other enzyme like AAC(6′)-I + AAC(3)-II. Although only speculative with the available data, the possibility that therapeutic nonequivalent generics could enhance enzymatic resistance deserves scientific testing [29, 31].

There are at least two hypotheses to explain the findings. First, Bau et al. described the X-ray crystal structure of amikacin [32], establishing that the spatial relationship depends on two bifurcated hydrogen (H) bonds that are necessary for the internal conformation of the amikacin molecule. The first H-bond is common with the molecule of kanamycin to control the A/B ring orientation but the second H-bond is required to define the conformational angles around the B/C ring junction. Any subtle change in the position of the second H-bond or its lack could reduce significantly the in vivo efficacy of amikacin. To test this hypothesis, one could compare simultaneously the chemical structure of innovator and generic by X-ray crystallography or NMR studies, however, the innovator is no longer available. Second, that impurities or different excipients might explain the failure of amikacin generics [33], but it is less likely because the process for semi-synthesis of amikacin from acylation of kanamycin A is a well-known process [34].

## Conclusions

In vitro susceptibility tests do not predict the in vivo efficacy of generic products of amikacin. Considering the potential impact on antimicrobial resistance of non-therapeutically equivalent generics, more studies comparing the molecular and chemical identity, as well as head-to-head studies in validated animal models of infection should be required before approval of generic amikacin products, although therapeutic equivalence will be difficult to establish without a gold standard (innovator product).

## Methods

### Antibiotics

All amikacin products were bought from reputable drugstores and handled following the instructions of each manufacturer. The innovator drug was included in all experiments as the gold standard [35]. Additionally, the reference powder (Sigma Aldrich, USA), a product not designed for clinical use, was used.

### Bacteria and media

*E. coli* SIG-1 (an ampicillin-resistant clinical isolate) was selected for in vitro and in vivo experiments. For susceptibility testing, *Pseudomonas aeruginosa* ATCC 27853 was used as control [36]. Culture media included trypticase soy broth and agar for in vivo studies and cation-adjusted Mueller–Hinton broth and agar for susceptibility testing (Becton–Dickinson, USA).

### Susceptibility testing

Minimal inhibitory (MIC) and bactericidal (MBC) concentrations of nine generic products, the reference powder and the innovator of amikacin were determined twice by broth microdilution following the Clinical Laboratory Standard Institute method [36]. To compare the in vitro potency, the differences between geometric means were assessed by Kruskal–Wallis (KW) test followed by Dunn’s multiple comparison test (GraphPad Prism 6.05) [37].

### Animal model

The University of Antioquia Animal Experimentation Ethics Committee approved the protocol. Six-week-old, 23–27 g, female murine-pathogen free mice of the strain Udea:ICR(CD-1) were used [38]. Mice were rendered neutropenic by injecting two intraperitoneal doses of cyclophosphamide (Cytoxan^®^, Bristol-Myers Squibb, Puerto Rico) given 4 days (150 mg/kg) and 1 day (100 mg/kg) before infection [39]. An intramuscular injection (0.1 mL) per thigh of a log-phase culture with ~7 log_10_ CFU of *E. coli* SIG-1 per mL was used. Two hours later (0 h), infected animals began a 24 h-treatment with each amikacin product (N ≥ 10 mice/product), allocating two animals per dose and using at least five total doses that ranged from no effect (1.5 mg/kg per day) to maximal effect (3072 mg/kg per day). Each dose was administered by the subcutaneous route (0.2 mL) every 6 h to optimize *f*C_max_/MIC and *f*AUC/MIC, the pharmacodynamic (PD) indices related to the efficacy of amikacin in mice and humans with normal renal function [40, 41]. Untreated infected control mice were sacrificed just after inoculation (−2 h), at the onset (0 h), and at the end of experiment (24 h), while treated mice were euthanized at 24 h.

To determine antibacterial efficacy, both thighs of each mouse were dissected under aseptic technique and homogenized independently in sterile saline (1:10). After serial dilutions and manual plating, the cultures were incubated for 18 h at 37 °C under air atmosphere before colony counting and data registration in a database (Microsoft Excel^®^, USA). In this model, one thigh weighs 1 g and the limit of detection is 100 CFU/thigh.

### Statistical analysis

For each total dose (independent variable), the net antibacterial effect (*E*, dependent variable) was calculated by subtracting the CFU/g obtained in thighs of infected mice from the 24 h untreated controls. Nonlinear regression of the dose–effect data from each product fitted to Hill’s model provided the primary PD parameters maximum effect (*E*_*max*_), effective dose killing 50 % of the *E*_*max*_ (*ED*_50_), and slope (*N*), as well as the secondary PD parameters bacteriostatic dose (*BD*) and the doses required to kill the first (*1LKD*) and second (*2LKD*) log of bacteria (SigmaPlot 12.3). To test the therapeutic equivalence, the magnitudes of these parameters were compared (each generic vs. the innovator) by curve fitting analysis (GraphPad Prism 6.05) as was described thoroughly elsewhere [13]. The quality of the nonlinear regressions was assessed by the adjusted coefficient of determination (adj.R^2^), the standard error of estimate (S_y|x_), the fulfillment of the assumptions of normality and homoscedasticity (constant variance), and the absence of multicollinearity (variance inflation factor). Accepting a 5 % chance for a type I error (α-error) and expecting residuals’ standard deviations ≤0.5 log, the treatment of 10 animals per product to compare nine generic products with the innovator confers 87 % power to reject the null hypothesis (H_0_: generics = innovator product) if the magnitude of the difference on antibacterial efficacy is >1 log_10_ CFU/g.

## Abbreviations

AAC(6′)-I: aminoglycoside 6′-*N*-acetyltransferase type I
AAC(3)-II: aminoglycoside 3-*N*-acetyltransferase type II
Adj. R^2^: adjusted coefficient of determination
ATCC: American Type Culture Collection
AUC: area under the curve concentration–time
BD: bacteriostatic dose
C_max_: the maximum concentration
CFA: curve fitting analysis
CFU: colony forming unit
CLSI: Clinical and Laboratory Standard Institute
E_max_: maximum effect
ED_50_: effective dose to kill 50 % of E_max_
MIC: minimal inhibitory concentration
MBC: minimal bactericidal concentration
N: Slope
NLR: non-linear regression
S_y|x_: standard error of the estimate
SE: standard error
1LKD and 2LKD: 1- and 2-log kill dose, respectively
